# From Body to Mind and Spirit: Qigong Exercise for Bereaved Persons with Chronic Fatigue Syndrome-Like Illness

**DOI:** 10.1155/2015/631410

**Published:** 2015-10-04

**Authors:** Jie Li, Jessie S. M. Chan, Amy Y. M. Chow, Lai Ping Yuen, Cecilia L. W. Chan

**Affiliations:** ^1^Renmin University of China, 59 Zhongguancun Street, 1007 Block D, Huixian Building, Haidian, Beijing 100872, China; ^2^Department of Social Work and Social Administration, The University of Hong Kong, Pokfulam, Hong Kong; ^3^Centre on Behavioral Health, The University of Hong Kong, Pokfulam, Hong Kong; ^4^International Association for Health and Yangsheng, Happy Valley, Hong Kong

## Abstract

Bereavement may bring negative impacts on the mind, body, and spiritual well-being of grieving persons. Some bereaved persons with chronic fatigue syndrome- (CFS-) illness experience a dual burden of distress. This study investigated the effects of bereavement on CFS-like illness by comparing bereaved and nonbereaved participants. It also adopted a random group design to investigate the effectiveness of Qigong on improving the well-being of bereaved participants. The Qigong intervention comprised 10 group sessions delivered twice a week for 5 weeks and home-practice for at least three times a week lasting 15–30 minutes each. The participants' fatigue, anxiety, and depression, quality of life (QoL), and spiritual well-being were measured at baseline and 3 months after treatment. The bereaved participants experienced significantly greater mental fatigue (16.09 versus 14.44, *p* = 0.017) and lower physical QoL (34.02 versus 37.17, *p* = 0.011) than their nonbereaved counterparts. After 3 months, the mental fatigue (−8 versus −4, *p* = 0.010) and physical fatigue (−10 versus −5, *p* = 0.007) experienced by intervention group had declined significantly, and improvements on their spirituality (14 versus −2, *p* = 0.013) and psychological QoL (8.91 versus 0.69, *p* = 0.002) scores exceeded those of the control group.

## 1. Introduction

The death of a loved one is considered to be one of the most distressing life experiences. Bereavement can profoundly affect various aspects of a person's well-being. First, it impairs bodily functions: it is associated with abnormal neuroendocrine changes [1], reduced immune response [2], and sleep disturbance [3, 4]. Stroebe et al. carried out an extensive review of the consequences of bereavement for health [5]. Most alarmingly, bereaved spouses face a higher risk of mortality, probably due to a higher morbidity rate among those with physical illnesses [5]. Second, various mental symptoms are associated with grief, the primary reaction to bereavement. Grieving individuals may not only be afflicted with anxiety, sadness, guilt, anger, and shame, but also experience a sense of disbelief, a preoccupation with memories of the deceased, the circumstances of his or her death, and the dying process, and an intense yearning or longing for the return of the deceased [6]. Moreover, bereavement may induce spiritual change: as a life crisis it can challenge one's assumptions about human existence [7]. Although the death of a loved one may incur great distress and impair one's spirituality [8], it may also trigger psychospiritual transformation and facilitate spiritual growth [9]. Therefore, researchers should pay more attention to ways of improving the spiritual well-being of the bereaved.

Given the wide scope of negative influences of bereavement, it is justifiable to hypothesize that grieving people are also at risk of chronic fatigue syndrome (CFS), which is associated with negative life events prior to the onset of illness [10]. Grieving persons share certain physiological/somatic symptoms with CFS patients, such as fatigue, sleep disturbance, exhaustion, somatic complaints, loss of appetite, and social withdrawal [5]. CFS is a condition characterized by a constellation of somatic and neurocognitive symptoms. It is defined as “clinically unexplained” fatigue lasting for at least six months and is considered to have no definite effective treatment [11]. CFS is associated with a constellation of physical and neurocognitive symptoms that may interfere with the patient's daily activities in family, work, and social life. However, due to the unknown etiology and lack of effective treatment for the condition, a large proportion of persons with it remain unrecognized and undertreated in the community. CFS-like illness is defined using criteria similar to those for CFS, but without confirmation by medical examination. Most persons with CFS-like illness have psychiatric disorders, for which they are often undertreated. CFS patients usually experience poor quality of life [12, 13] and report greater psychological distress and lower functional well-being than their healthy peers [13, 14].

As bereaved persons with CFS bear the double burden of grief and chronic fatigue, researchers and clinicians should aim to identify techniques to help them adjust and improve their well-being. To date, several methods have been implemented to assist bereaved persons, such as pharmacotherapy [15], psychotherapy [16], and various combinations of pharmacotherapy, psychotherapy, and psychoeducation [17]. However, researchers have shown that these types of intervention have only minor positive effects on the bereaved [16, 18–21]. Thus, it is necessary to develop alternative forms of bereavement intervention. In a recent review, a novel body-mind intervention was found to outperform two major traditional forms of bereavement intervention: emotional expression and education [22]. In accordance with this trend, Qigong, a Chinese body-mind exercise, may provide new insight into the situation.

Qigong is an ancient Eastern self-healing technique.* Qi* means “vital internal energy”;* gong* denotes “practice or skill.” According to Qigong practitioners, the body, mind, and spirit are sufficiently intertwined that physical adjustments can result in mental, emotional, and spiritual changes. Qigong can facilitate the regulation of the mind, body, and breathing. It focuses on the balance between yin and yang, as well as regulating the circulation of Qi within the meridian system (Qi vital energy channel) to foster a sense of somatic harmony that consequently helps in bringing about changes in mood and spiritual well-being. Qigong can be used as a self-care technique to buffer stress and improve one's quality of life and spiritual state [23]. A systematic review showed that Qigong reduces stress and anxiety [24]. Recently, Qigong has been found to have beneficial effects on persons with CFS-like illness, who have shown limited clinical reaction to pharmaceutical treatments. It can reduce patients' fatigue, anxiety, depressive symptoms, and sleep disturbance, improve their mental functioning, and even alter their telomerase activity [25–27].

Given the promising effects of Qigong exercise on CFS-like illness, it is postulated that Qigong should have positive effect on bereaved persons with CFS-like illness. Moreover, there are several advantages of using Qigong to assist bereaved persons. First, current popular interventions with bereaved persons are mainly psychotherapies that address the emotional and psychosocial aspects of bereavement. Qigong, based on the integrated body-mind-spirit framework, is likely to have a holistic effect on the multidimensional reactions of bereavement. Second, most current bereavement interventions are provided by mental health professionals and accessibility may become a problem as professional resources are often limited. As a result, some people may not obtain sufficient or timely help [5]. Third, although current interventions may have positive short-term effects, they may be difficult to sustain in the long term if treatment ends [19]. Self-help methods such as Qigong exercises can be used to supplement and sustain these positive outcomes through continuous practice.

The aims of this study were to investigate the effects of bereavement on individuals with CFS-like illnesses and to explore the potential of Qigong exercises to assist bereaved persons with CFS-like illness. To the best of our knowledge, no previous study has applied Qigong to treat this group of people or evaluated the effectiveness of Qigong as an alternative approach to assisting bereaved persons. The data were drawn from a larger research program on CFS-like illness with a random controlled group design, which investigated the effects of Qigong on multiple aspects of well-being, such as physical symptoms, mental health, spirituality, and quality of life.

## 2. Methods

### 2.1. Study Design

The study was part of a larger prospective randomized waiting-list controlled study. More details of the recruitment and randomization processes can be found in a previous report [27]. After providing informed consent, the participants in both groups completed the questionnaires first at baseline and again 3 months after intervention. The intervention comprised a 10-session Qigong course plus 15–30-minute self-practice sessions at least three times per week. The ten Qigong sessions (*wu xing ping heng gong*) lasted for 2 hours each and were delivered twice a week for 5 weeks by an experienced Daoist Qigong master. Each session comprised a brief introduction of the basic theories of traditional Chinese medicine (the concepts of Qi, yin-yang, five elements, and meridian system), precautions on practicing Qigong exercise, and answering any questions or concerns raised by the participants (45 minutes), followed by warm-up movements including gentle movements or body stretching (15 minutes) and a one-hour session of Qigong exercise training. More details of the description of Qigong exercise can be found in the intervention section in a previous report [27]. The participants in the control group were advised to maintain their usual lifestyles and refrain from joining Qigong classes for the duration of the study. The same intervention was delivered to the participants in the control group after the final outcome measurements had been collected.

### 2.2. Study Participants

Between October 5 and October 14, 2010, the study was publicized in the media and 1441 Chinese adults who claimed to experience fatigue symptoms were recruited as potential participants. The volunteers were screened using an online questionnaire based on the CFS diagnostic criteria established by the US Center for Disease Control and Prevention [11], but without further medical examination. Volunteers were considered to suffer from a CFS-like illness if they met the following two inclusion criteria: (1) unexplained, persistent fatigue that could not be relieved by rest and that resulted in a significant reduction in previous activity levels and (2) having four or more of the following symptoms for 6 or more months: (a) impaired memory or concentration, (b) postexertional malaise (extreme and prolonged exhaustion following physical or mental activity), (c) unrefreshing sleep, (d) muscle pain, (e) multijoint pain, (f) headaches of a new type, (g) sore throat, and (h) tender lymph nodes. Based on a medical history checklist in the online questionnaire, the persons with any history of cancer, sleep apnea, hypothyroidism, narcolepsy, hepatitis B or C virus infection, severe obesity, mental disorders, including schizophrenia, major depressive disorder, and bipolar disorder, and alcohol or other substances' abuse were excluded. As CFS mainly affects young adults, patients over 60 were also excluded, to minimize the possibility that CF was experienced due to ageing and/or other comorbid chronic conditions.

Only 154 participants with CFS-like illness were recruited into the study. Eighteen participants dropped out. Of the remaining 136 participants, 46 had been bereaved within the previous 2 years. They were randomly assigned to the Qigong group (*n* = 22) and the control group (*n* = 24).

### 2.3. Measures

Demographic data including age, gender, employment status, education level, marital status, religion, and bereavement experience within 2 years were collected through a questionnaire.

The severity of fatigue was assessed using the Chalder Fatigue (CF) scale, which is a 14-item self-rating scale. It has been shown to be reliable and valid. Respondents score the items using a Likert-type response scale where 0 is none, 1 is better than usual, 2 is no more than usual, 3 is worse than usual, and 4 is much worse than usual. The CF scale shows a high degree of internal consistency, with a principal components analysis supporting the notion of a two-factor solution (physical and mental fatigue). Items 1–8 measure physical fatigue, and items 9–14 measure mental fatigue. A total score is obtained by summing all of the item scores [28]. The Chinese version of CF scale has been validated and found to be reliable among Chinese adults in the general population [29].

Mental health was assessed using the Hospital Anxiety and Depression Scale (HADS), which comprises two subscales: HADS-A (7 items) and HADS-D (7 items). HADS-A and HADS-D measure the respondents' anxiety and depression, respectively, in the previous week [30]. HADS has a high internal consistency for both anxiety (Cronbach's *α* = 0.93) and depression (Cronbach's *α* = 0.90). In addition, both the anxiety subscale and the depression subscale have a high test-retest reliability (*r* = 0.89 and *r* = 0.92, resp.). The Chinese version of HADS has also been validated with good internal consistency and test-retest reliability [31].

Quality of life was assessed using the Short Form Health Survey (SF-12), a 12-item questionnaire with separate subscales for physical health and mental health. Higher scores signify higher functioning [32]. The SF-12 has been translated into Chinese and validated for use in Hong Kong [33].

Spiritual well-being was assessed using the “spirituality” subscale of the Body-Mind-Spirit Well-being Inventory (BMSWBI-S) [34], a 13-item questionnaire with three distinct components: tranquility, disorientation, and resilience. Spirituality is measured by evaluating the patient's core values and philosophy and the extent to which he or she regards life as having meaning. The measure has proven to bear sound validity, high internal consistency, and test-retest reliability when used with Chinese respondents [34].

### 2.4. Statistical Analysis

First, bereaved and nonbereaved participants were compared in terms of fatigue severity, mental health, spirituality, and health-related quality of life using chi-square test for the categorical data and a* t*-test for the continuous data. To confirm that the intervention group and control group were comparable, demographic data and outcome variables at baseline for the intervention and control groups were compared using a chi-square test and the Mann-Whitney test, due to the small sample size. The effects of practicing Qigong were examined by comparing the outcome variables at baseline with those observed 3 months after intervention. Qigong group and control group were compared in terms of their preintervention score, postintervention score, and the changes between before and 3 months after treatment (T1–T0). All data analysis was conducted using the Statistical Package for the Social Sciences 18.0.

## 3. Results

As shown in Table 1, the bereaved persons with CFS-like illness exhibited significantly greater mental fatigue (16.09 versus 14.44, *p* = 0.017) and received significantly lower scores for the physical-health component of the SF-12 (34.02 versus 37.17, *p* = 0.011) than their nonbereaved counterparts.

The Qigong group and the control group were comparable in terms of both their demographic characteristics and their scores for the baseline outcome variables of fatigue, mental health, and health-related quality of life (see Tables 2 and 3).

After 3 months of Qigong intervention, the Qigong group reported a significant decrease in total fatigue (−17 versus −9, *p* = 0.007) and mental fatigue (−8 versus −4, *p* = 0.010) and significant improvements in their psychological quality of life (8.91 versus 0.69, *p* = 0.002) compared to the control group.

Compared with the control group, the Qigong group experienced a significant reduction in physical fatigue after the Qigong intervention (−10 versus −5, *p* = 0.007). However, no significant difference in physical quality of life was observed between the two groups (2.66 versus 3.79, *p* = 0.451).

The improvement in spirituality score from baseline to 3 months after intervention (T1–T0) was also found to be significant (14 versus −2, *p* = 0.013). However, the spirituality score of the Qigong group at T1 did not improve significantly, compared with that of the control group (72 versus 68, *p* = 0.183). No significant changes were observed in anxiety, and the change in depression was only marginally significant. No adverse events were observed.

## 4. Discussion

Bereaved participants with CFS-like illness in this study had significantly higher mental fatigue scores and lower physical functioning than did the nonbereaved participants. This result underlines the negative influence of bereavement on well-being. It is also consistent with an early assumption that stressful events such as bereavement are a potential cause of CFS [10, 35].

The study's findings suggest that Qigong is an effective aid for bereaved persons with CFS-like illness. Comparing the Qigong group with the control group confirmed that Qigong improves the health of bereaved persons. Having practiced Qigong for a period, the participants reported a significant decrease in negative physical symptoms. This finding is consistent with the results of previous studies, which have found similar effects of Qigong on physical health among patients with chronic illnesses [36] and with CFS-like illnesses [25–27]. This study extends these findings to bereaved persons. It should be noted that the change in the participants' average score for the physical component of the SF-12 was not significant. Long-term Qigong practice may be necessary to improve physical functioning [25].

The data also indicated that Qigong reduced the patients' mental fatigue and improved mental functioning, although no significant changes were observed in their depression or anxiety. Similarly, the authors of an earlier study with a small sample (*n* = 50) found that Qigong improved psychological health but did not significantly decrease the depression score [36]. However, another large scale study (*n* = 137) showed that Qigong had an antidepressive effect but did not significantly reduce anxiety symptoms for persons with CFS-like illness compared with a control group [27]. In a later study (*n* = 150) with a longer intervention (16 Qigong sessions and daily self-practice sessions lasting at least 30 minutes each), Qigong was found to significantly reduce the anxiety and depressive symptoms reported by patients with CFS-like illness [26]. The marginally significant improvement in depressive symptoms (*p* = 0.072) in the current study was probably due to small sample size (*n* = 46). Whether Qigong is effective in alleviating depression and anxiety symptoms in bereaved persons deserves further examination using a larger sample size.

Although the spiritual well-being of bereaved persons is an area of interest for both researchers and clinicians, few related interventions have been proposed or tested. The results of this study indicate that Qigong not only improves the physical fatigue, mental fatigue, and mental functioning of bereaved persons but also enhances their spiritual well-being. As it both reduces negative physical, mental symptoms and improves psychological quality of life and spiritual state, Qigong offers a promising complementary and alternative therapy for bereaved persons.

However, the limitations of the study should be acknowledged. First, the use of a small sample restricted to those with symptoms of severe mental and physical fatigue may reduce the generalizability of the findings to the larger bereaved population. Second, all of the outcome variables were self-reported and may thus be subject to individual bias. To validate the findings, the research should be repeated with a larger sample size and biological markers as outcomes. Third, the mechanisms by which Qigong influences patient health were not examined. It is possible that Qigong acts as a stress reduction strategy to regulate body and mind to achieve a state of homeostasis and promote self-healing [37]. This process deserves further exploration.

Despite the above limitations, the study's findings suggest that Qigong has the capacity to assist bereaved persons experiencing physical and psychological disturbances. Compared with other treatments, such as medication and psychological counseling, Qigong has distinct advantages for bereaved people, as it is easy to learn and is self-administered and has few side-effects. It may thus encourage patient compliance with treatment and can be used as a self-help method in the long term. Moreover, Qigong combines physical exercises with mindful practice and enhances physical, mental, and spiritual well-being.

In conclusion, the use of Qigong as a self-care practice can reduce both physical and mental fatigue and improve the psychological quality of life and spiritual well-being of bereaved persons with CFS-like illness. Researchers and clinical practitioners should thus pay more attention to Qigong as a potential aid for bereaved persons. As the sample used in this study was small, more extensive and rigorous research is needed to confirm our findings.

## Figures and Tables

**Table 1:** Comparison of bereaved and nonbereaved participants with CFS-like illness at baseline (*n* = 136).

	Bereaved (*n* = 46)	Nonbereaved (*n* = 90)	*p* ^*∗*^
	Mean (SD)	Mean (SD)	
CF total score	40.87 (6.26)	38.94 (6.25)	0.092
CF: physical score	24.78 (4.16)	24.50 (3.65)	0.684
CF: mental score	16.09 (3.33)	14.44 (3.93)	0.017

HADS			
Anxiety	10.96 (2.17)	10.93 (2.25)	*0.954 *
Depression	9.13 (2.23)	9.27 (2.06)	*0.718 *

SF-12			
SF-PSC	34.02 (6.00)	37.17 (7.11)	*0.011 *
SF-MSC	33.22 (9.50)	32.80 (9.64)	*0.807 *

Spirituality			
Total score	68.74 (25.11)	67.52 (25.10)	*0.790 *
Tranquility	25.39 (10.64)	24.40 (10.37)	*0.602 *
Disorientation	24.76 (11.66)	24.81 (12.08)	*0.982 *
Resilience	18.11 (6.73)	17.93 (6.19)	*0.880 *

CF: Chalder Fatigue; ^*∗*^ *t*-test.

HADS: Hospital Anxiety and Depression Scale (score interpretation for each subscale: 0–7: normal; 8–10: mild; 11–14: moderate; 15–21: severe).

PCS: physical-component summary; MCS: mental-component summary.

**Table 2:** Demographic characteristics for bereaved participants with CFS-like illness (*n* = 46).

Demographics	Intervention (*n* = 22)		Control (*n* = 24)		*p* ^*∗*^
	Median (range)	*N* (%)	Median (range)	*N* (%)	
Age (years)	46 (23–52)		45 (32–51)		0.508
Gender					
Female		19 (86.4%)		21 (87.5%)	
Employment					0.670
Full-time		16 (72.7%)		20 (83.3%)	
Part-time		2 (9.1%)		0	
Housewife		2 (9.1%)		2 (8.3%)	
Unemployed		1 (4.5%)		1 (4.2%)	
Other		1 (4.5%)		1 (4.2%)	
Education					0.594
Forms 1 to 5		10 (45.5%)		12 (50.0%)	
Forms 6 to 7		2 (9.1%)		3 (12.5%)	
Tertiary/university		9 (40.9%)		6 (25.0%)	
Master's level or above		1 (4.5%)		3 (12.5%)	
Marital status					0.336
Single		3 (13.6%)		7 (29.2%)	
Married/cohabiting		15 (68.2%)		15 (62.5%)	
Divorced/separated		4 (18.2%)		2 (8.3%)	
Religion					
Yes		6 (27.3%)		9 (37.5%)	0.460
Bereavement within 2 yrs					0.559
Spouse		1 (4.5%)		0	
Sibling		2 (9.1%)		3 (12.5%)	
Parents		10 (45.5%)		8 (33.3%)	
Others		9 (40.9%)		13 (54.2%)	

^*∗*^Chi-square test of categorical variable and Mann-Whitney test of continuous variable.

**Table 3:** Comparison of bereaved participants with CFS-like illness in Qigong group and control group (*n* = 46).

	Qigong group (*n* = 22)	Control group (*n* = 24)	*p* ^*∗*^
	Median (range)	Median (range)	
CF total score			
Before (T0)	41.5 (28–53)	40 (31–53)	0.956
After (T1)	21 (8–34)	37 (11–50)	0.003
T1–T0	−17 (−35–−6)	−9 (−22–8)	0.007
CF: physical score			
Before (T0)	24 (15–32)	24.5 (19–32)	0.791
After (T1)	16 (4–22)	21 (9–32)	0.002
T1–T0	−10 (−23–0)	−5 (−14–6)	0.007
CF: mental score			
Before (T0)	17 (12–21)	16 (7–22)	0.903
After (T1)	6 (2–17)	13 (2–21)	0.011
T1–T0	−8 (−16–0)	−4 (−9–5)	0.010
HADS			
Anxiety			
Before (T0)	11 (8–14)	11 (6–16)	*0.929 *
After (T1)	8 (2–12)	9 (2–18)	*0.259 *
T1–T0	−3 (−10–1)	−2 (−12–11)	*0.376 *
Depression			
Before (T0)	8 (5–12)	10 (5–15)	*0.051 *
After (T1)	7 (3–12)	11 (2–17)	*0.016 *
T1–T0	−1 (−5–4)	0 (−7–8)	*0.072 *
Spirituality			
Total score			
Before (T0)	63 (8–116)	78.5 (16–118)	*0.169 *
After (T1)	72 (61–116)	68 (29–118)	*0.183 *
T1–T0	14 (−37–62)	−2 (−37–36)	*0.013 *
SF-12			
SF-PCS			
Before (T0)	34.83 (25.55–45.01)	31.60 (21.47–46.57)	*0.141 *
After (T1)	40.00 (22.79–53.15)	35.82 (24.84–49.70)	*0.511 *
T1–T0	2.66 (−12.67–17.97)	3.79 (−1.76–22.19)	*0.451 *
SF-MCS			
Before (T0)	31.49 (12.20–49.65)	35.63 (11.09–49.14)	*0.582 *
After (T1)	45.54 (28.88–57.16)	36.59 (16.43–54.25)	*0.002 *
T1–T0	8.91 (−2.14–30.94)	0.69 (−23.07–16.34)	*0.002 *

CF: Chalder Fatigue; HADS: Hospital Anxiety and Depression Scale; PCS: physical-component summary; MCS: mental-component summary; ^*∗*^Mann-Whitney test.
